# From rest to meaning: default mode network neuroscience as a design constraint for early childhood education

**DOI:** 10.3389/fpsyg.2026.1815826

**Published:** 2026-07-29

**Authors:** Kijoo Cha

**Affiliations:** Graduate School of Education, Ewha Womans University, Seoul, Republic of Korea

**Keywords:** default mode network, early development, early childhood education, early learning, narrative, wakeful rest

## Abstract

The brain’s default mode network (DMN) supports internally oriented processes implicated in meaning-making, autobiographical memory, narrative comprehension, and social understanding. Despite rapid growth in DMN research, its relevance to early learning remains under-specified in educational discourse—particularly in competitive contexts where long-standing early childhood priorities (e.g., play, rest, and child agency) can be squeezed by intensifying early academic pressure. This structured integrative review of neuroscientific and developmental literature (1997–January 2026) synthesizes (1) DMN functions relevant to learning, meaning-making, and socio-emotional development, (2) developmental features of DMN organization from infancy to the preschool years that constrain educational expectations, and (3) conservative educational implications framed as context-level principles for organizing everyday learning conditions rather than as neural prescriptions. Across studies, early resting-state organization and some DMN-related nodes or motifs are detectable in infancy, whereas a complete adult-equivalent DMN, long-range connectivity, hub organization, and coordination with attention and control systems emerge gradually and remain strongly state-sensitive in the first years of life. A recurring implication concerns mode switching: externally focused engagement and internally oriented integration rely on partially competing large-scale networks, highlighting the developmental logic of routines that interleave guided activity with periods of child-initiated play, quiet wakeful rest, and narrative revisiting. The review concludes by articulating DMN-informed design principles for early childhood settings: balanced time structures that protect downtime, narrative-rich interaction, emotionally secure co-regulation, and constructive solitude within a supportive social ecology. It also outlines priorities for future work linking such contextual organization to learning and well-being without treating DMN activity as a direct instructional target.

## 1 Introduction

Interest in the Default Mode Network (DMN) rose sharply after converging PET and fMRI evidence showed reliable task-induced deactivations in midline and parietal regions during externally focused cognition, reframing the baseline state as an organized mode of brain function rather than statistical “noise” (Shulman et al., 1997; Raichle et al., 2001). Intrinsic functional connectivity work then demonstrated that these regions form a coherent resting-state network (Greicius et al., 2003), and subsequent syntheses moved beyond a “task-negative” label toward models in which DMN nodes contribute to autobiographical memory, prospection, semantic integration, social cognition, and spontaneous thought (Buckner et al., 2008; Smallwood et al., 2021). Recent integrative accounts propose that the DMN helps bind memory, language, and semantic representations into an ongoing internal narrative that supports subjective continuity and aspects of selfhood (Menon, 2023).

Despite rapid growth in the neuroscience of the default mode network (DMN), early childhood educators and policymakers rarely reference DMN concepts explicitly when debating how to balance child-initiated play with increasingly early academic expectations (e.g., foundational literacy and numeracy instruction) in birth-to-school-entry settings. This gap matters because early childhood education has long valued play, rest, and child agency, yet these elements can be squeezed by “push-down” pressures and expanding adult-directed instruction in competitive educational climates. Illustratively, kindergarten classrooms in the United States between 1998 and 2010 showed decreases in child-selected activities alongside increases in teacher-directed instruction (Bassok et al., 2016), and play-based approaches are frequently described as being constrained under rising academic pressure in early education contexts (Bodrova et al., 2023). Comparable pressures are also visible in East Asian contexts where school readiness and early academic preparation can begin well before formal schooling. For example, in South Korea, a government trial survey of private education expenses for children under six indicated that nearly half (47.6%) of children under six participated in some form of private education. In these households, while spending on academic/general-subject and essay-related instruction was substantially higher than that on arts or sports, the total national expenditure across all categories of private education reached over 815 billion KRW over a three-month period (Statistics Korea and Ministry of Education, 2025)—approximately US $598 million at the 2024 annual-average exchange rate.

This situation illustrates a broader policy concern: in highly competitive educational ecologies, academic preparation can enter early childhood routines in ways that compress time for play, rest, narrative revisiting, and child-initiated activity. The relevance of the DMN begins at precisely this point: when early learning is organized almost exclusively around externally directed performance, children may have fewer opportunities for the internally oriented integration through which they revisit, consolidate, and make meaning from experience. In this context, making DMN concepts legible to parents, educators, and policymakers can help clarify why protecting cycles of engagement and integration is developmentally coherent—but only if the translation remains conservative. Translational work is vulnerable to overreach: claims of the form “the DMN is involved in X” can be misread as “teachers should train the DMN,” even when the most defensible inference is simply that certain learning conditions plausibly support cognitive mechanisms associated with X. For this reason, critiques of educational neuroscience repeatedly emphasize treating neural evidence as constraints and plausibility arguments rather than as classroom prescriptions (Bruer, 1997; Howard-Jones, 2014; Bowers, 2016). Accordingly, this review adopts a conservative translational stance: DMN findings are used to clarify cognitive and developmental constraints and to interpret where well-established early childhood practices already align with those constraints, rather than to propose “DMN-based” techniques.

A second gap concerns developmental specificity. Much of what is popularly said about the DMN derives from adult and adolescent research, yet developmental neuroimaging shows that early DMN organization differs meaningfully from adult patterns. DMN-like fragments can be detected early, but long-range integration and stable interactions with attention/control networks mature gradually across infancy, toddlerhood, and the preschool years. Moreover, many infant resting-state datasets are collected during natural sleep to reduce motion, and sleep itself alters functional connectivity—including within the DMN—complicating direct comparison with adult awake rest. Accordingly, educational interpretation must begin with a developmentally grounded summary of what is known about early DMN maturation and its measurement caveats.

The purpose of this paper is threefold: (a) to provide an accessible but academically grounded overview of what the DMN is and why it matters for cognition and learning; (b) to synthesize evidence on DMN development from infancy through preschool; and (c) to translate convergent findings into a small set of defensible educational design principles—principles that emphasize rhythms of engagement and integration, narrative meaning-making, and emotionally secure contexts—while explicitly stating limits of inference.

## 2 Methods

### 2.1 Design and data search

This study employed a structured integrative literature review design aimed at synthesizing neuroscientific evidence on the DMN and translating those findings into conservative implications for early childhood education (ECE) (birth to school entry). Because the topic spans cognitive neuroscience, developmental neuroscience, developmental psychology, and ECE, the review combined a systematically informed search strategy with thematic synthesis across heterogeneous literatures. The review was not designed as a quantitative meta-analysis of homogeneous intervention studies; instead, it used systematic search documentation and thematic integration to support conservative cross-disciplinary synthesis.

Searching, screening, extraction, and synthesis were conducted between October 2025 and February 2026. Database searches were last updated in January 2026; subsequent work involved screening completion, extraction review, citation checking, source classification, and synthesis. Databases searched included PubMed/MEDLINE, Web of Science, Scopus, PsycINFO, ERIC, and Google Scholar. Reference lists of key DMN syntheses and DMN discussions in education were also screened to identify additional relevant publications.

The search covered publications from 1997 through January 2026. The start date was selected because the modern DMN construct emerged from late-1990s PET and fMRI work identifying consistent task-related deactivations (e.g., Shulman et al., 1997; Raichle et al., 2001). Search strings were adapted for each database. A core Boolean structure was: “default mode network” OR “DMN” AND (infant OR infancy OR neonate OR toddler OR preschool OR “early childhood” OR “0–6 years” OR child*) AND (education OR learning OR play OR narrative OR language OR “self-regulation” OR “executive function” OR creativity OR imagination OR rest OR consolidation). For Google Scholar, the first 200 results per query (sorted by relevance) were screened to support citation tracing. Supplementary queries targeted adjacent constructs (e.g., resting-state functional connectivity in infants; autobiographical memory development; narrative comprehension neuroimaging; wakeful rest and consolidation; dialogic reading; elaborative reminiscing; screen exposure; sleep; stress). Backward and forward citation chaining was used for highly cited sources to ensure coverage of foundational and recent work. Although the formal search window began in 1997, one seminal pre-1997 source identified through citation chaining, Coplan et al. (1994), was retained for conceptual framing.

### 2.2 Eligibility criteria

#### 2.2.1 Inclusion criteria

Only publications written in English were included. Non-English articles were excluded due to feasibility constraints. Studies were included if they met one or more of the following criteria:

1. *Peer-reviewed empirical studies* using neuroimaging or closely related measures relevant to large-scale network organization (e.g., resting-state fMRI, task-based fMRI (including naturalistic paradigms), PET, resting-state fNIRS; and EEG/ERP when explicitly interpreted in relation to DMN-level organization or network interactions)
2. Peer-reviewed meta-analyses or systematic reviews synthesizing DMN-related evidence
3. Peer-reviewed theoretical, methodological, or integrative articles/reviews providing foundational or widely cited frameworks relevant to DMN function, development, large-scale network interactions or educational translation
4. Studies examining:

- DMN function in typically developing adults or adolescents (for conceptual grounding of DMN-linked constructs),
- DMN development in neonates/infants, toddlers, and preschool-aged children (0–6 years),
- Cognitive/affective constructs strongly associated with DMN (e.g., autobiographical memory, semantic processing, narrative comprehension, self-referential thought/mind wandering, creative ideation, social cognition/mentalizing),
- Developmentally grounded, education-relevant contexts or practices plausibly engaging these constructs (e.g., narrative interaction, play, reflection/constructive solitude, and classroom/home routines that allow transitions between engagement and integration).

During synthesis, priority was given to comprehensive reviews and meta-analyses, larger developmental samples, and empirical studies with clearer reporting of behavioral state, motion handling, and analytic approach.

#### 2.2.2 Exclusion criteria

The following were excluded: 1) conference abstracts without full peer-reviewed publication; 2) non-peer-reviewed editorials, opinion essays, or commentary; 3) commercial publications, or non-scholarly media articles; 4) animal-only studies; and 5) for empirical studies, reports with insufficient methodological detail to permit interpretation (e.g., unclear sample size, unspecified modality, or unclear analytic approach). Clinical or high-risk samples were not automatically excluded; however, they were not treated as primary evidence for normative DMN development unless the study directly informed developmental network organization or clarified interpretive constraints. Such sources were coded as *contextual* or *non*-*core* when their primary focus was clinical prediction, injury, disorder, or methodological validation rather than typical DMN maturation (See Supplementary Table S2).

### 2.3 Screening, corpus construction, and source classification

All screening and extraction steps were completed by a single reviewer. To reduce selection bias, eligibility decisions and extracted summaries were revisited after an interval using the predefined criteria, and citation chaining was used to check coverage of foundational and recent work. The absence of independent double screening is acknowledged as a limitation.

A total of 1,209 records were identified across databases. After duplicate removal (*n* = 269), 940 unique records remained and were screened at the title/abstract level for relevance to (a) DMN neuroscience, (b) early development (0–6 years), and/or (c) educationally relevant DMN-linked constructs. At this stage, 420 records were excluded. Full-text reports (*n* = 520) were retrieved and assessed for eligibility, and 395 were excluded due to irrelevance (e.g., clinical/patient-only samples without clear relevance to normative development or education-relevant constructs), non-peer-reviewed status, or insufficient methodological detail. The original database search and citation-chaining process yielded 125 eligible publications, initially organized into a 70-source core thematic synthesis set and a 55-source eligible non-core/contextual set. During revision, bounded supplementary citation checks were conducted in areas identified as coverage gaps: neonatal and perinatal functional connectivity, Developing Human Connectome Project (dHCP) and Baby Connectome Project (BCP) publications, and broader neurodevelopmental mechanisms relevant to DMN maturation, including synaptic overproduction, pruning/refinement, myelination, and protracted maturation of heteromodal association cortex. These supplementary checks added 18 eligible sources, producing a final documented corpus of 143 sources: 87 sources in the revised core thematic synthesis set, documented in Supplementary Table S1, and 56 eligible non-core/contextual sources, documented in Supplementary Table S2.

Sources were re-coded using a directness-and-translation rubric. Sources were assigned to the revised core thematic synthesis set when they met at least one of four roles: 1) direct evidence on DMN/default-network development in the birth-to-six age range or perinatal period; 2) direct DMN evidence for focal cognitive-affective constructs central to the review, including autobiographical memory, semantic integration, narrative comprehension, mentalizing, creativity, mind wandering, wakeful rest, and mode switching; 3) major theoretical, methodological, or perspective frameworks necessary for interpreting DMN function, developmental measurement constraints, cross-network dynamics, or educational translation; or 4) high-leverage developmental or educational evidence that served as primary behavioral support for a practice-level claim explicitly mapped to a DMN-linked construct. Sources in category 4 were not treated as direct DMN evidence and were used only to support DMN-consistent, construct-aligned educational principles.

**Figure 1:**
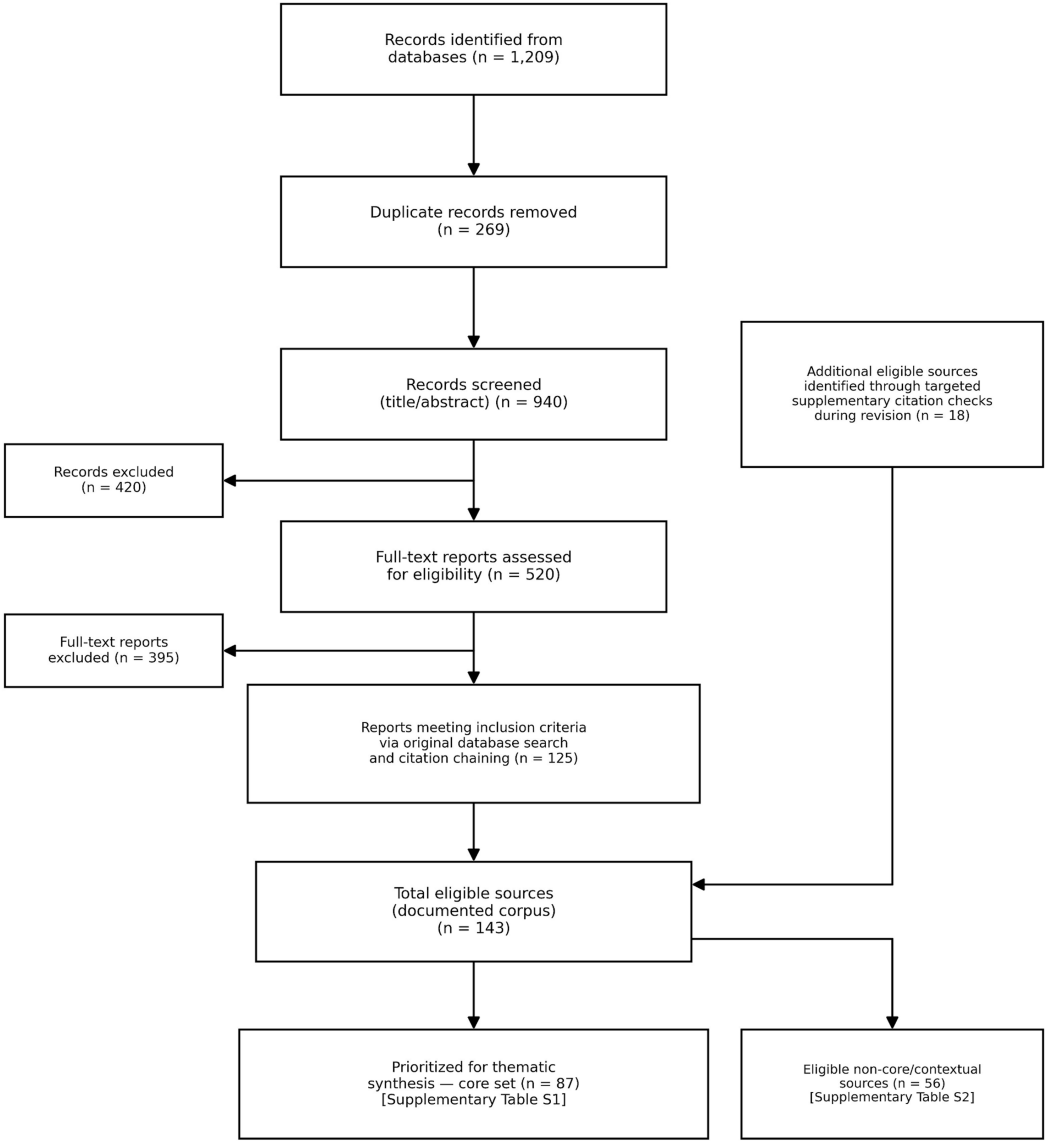
PRISMA-style flow diagram documenting identification, screening, eligibility assessment, supplementary citation checking, and the final documented corpus used in this structured integrative review.

Supplementary Table S1 is therefore a tiered synthesis list, not a list of studies that all provide direct empirical DMN evidence. Supplementary Table S2 documents eligible sources retained for contextual, methodological, clinical/high-risk, age-adjacent, redundant/corroborative, policy/background, or indirect practice-level support. When Supplementary Table S2 sources are cited in the manuscript, they are not used as primary support for DMN-specific claims. Reporting-guideline and policy/statistical sources were used only for reporting or background framing and were not treated as synthesized empirical or theoretical sources. The PRISMA-style flow diagram was used as a reporting aid to document identification, screening, eligibility assessment, supplementary citation checking, and final corpus construction; it was not intended to imply formal PRISMA compliance or quantitative aggregation of homogeneous intervention studies (Page et al., 2021). The source-identification and corpus-construction process is summarized in Figure 1.

### 2.4 Data extraction, coding, and thematic synthesis

Because the corpus integrated empirical neuroimaging studies, meta-analyses, systematic reviews, theoretical/perspective articles, methodological papers, and developmental/educational intervention studies, a single formal risk-of-bias instrument was not appropriate across the full evidence base. Instead, sources were coded for methodological interpretability and translational directness. Coding fields in Supplementary Tables S1, S2 include publication type/design, modality/method, population/age focus, sample information, section linkage, synthesis axis, main construct/focus, evidence category, relevance to the DMN framework, and role in thematic synthesis or classification rationale. For empirical sources, methodological interpretability reflected factors such as sample size, behavioral-state handling, motion control, modality, and analytic clarity. Translational directness reflected whether a source provided direct DMN evidence, construct-level evidence, developmental or educational behavioral evidence, methodological context, or background framing.

Data were synthesized using a thematic integrative approach organized around three axes: DMN function in adult cognition as conceptual grounding; DMN developmental trajectory from infancy through preschool, including state-dependent measurement issues; and translational constraints relevant to education, including mode switching, wakeful rest/offline processing, narrative meaning-making, emotionally secure co-regulation, constructive solitude, social interaction, and play.

The translational logic used here is construct mapping rather than direct neural prescription. DMN research was used to identify cognitive and developmental constraints, such as internally oriented integration, autobiographical memory, narrative situation-model construction, social mentalizing, wakeful rest, and switching between externally and internally oriented modes. Developmental and educational literatures were then used to identify practices that support these constructs behaviorally, such as elaborative reminiscing, dialogic reading, socio-dramatic play, emotionally secure co-regulation, protected low-demand rest, and constructive solitary or collaborative play. Educational implications were framed as DMN-consistent design principles only when the practice was behaviorally supported and theoretically aligned with DMN-linked functions. Unless a study directly linked a practice to DMN structure, activation, or connectivity, the implication was not described as DMN-proven. This approach was adopted to avoid level-mixing between neural findings and classroom prescriptions: neuroscientific studies were used to clarify mechanisms and developmental constraints; educational and developmental studies were used to support practice-level recommendations; and all implications were stated at the most conservative level supported by the relevant evidence category.

## 3 Results

### 3.1 What the DMN is, what it supports, and why it matters for learning and development

**Figure 2:**
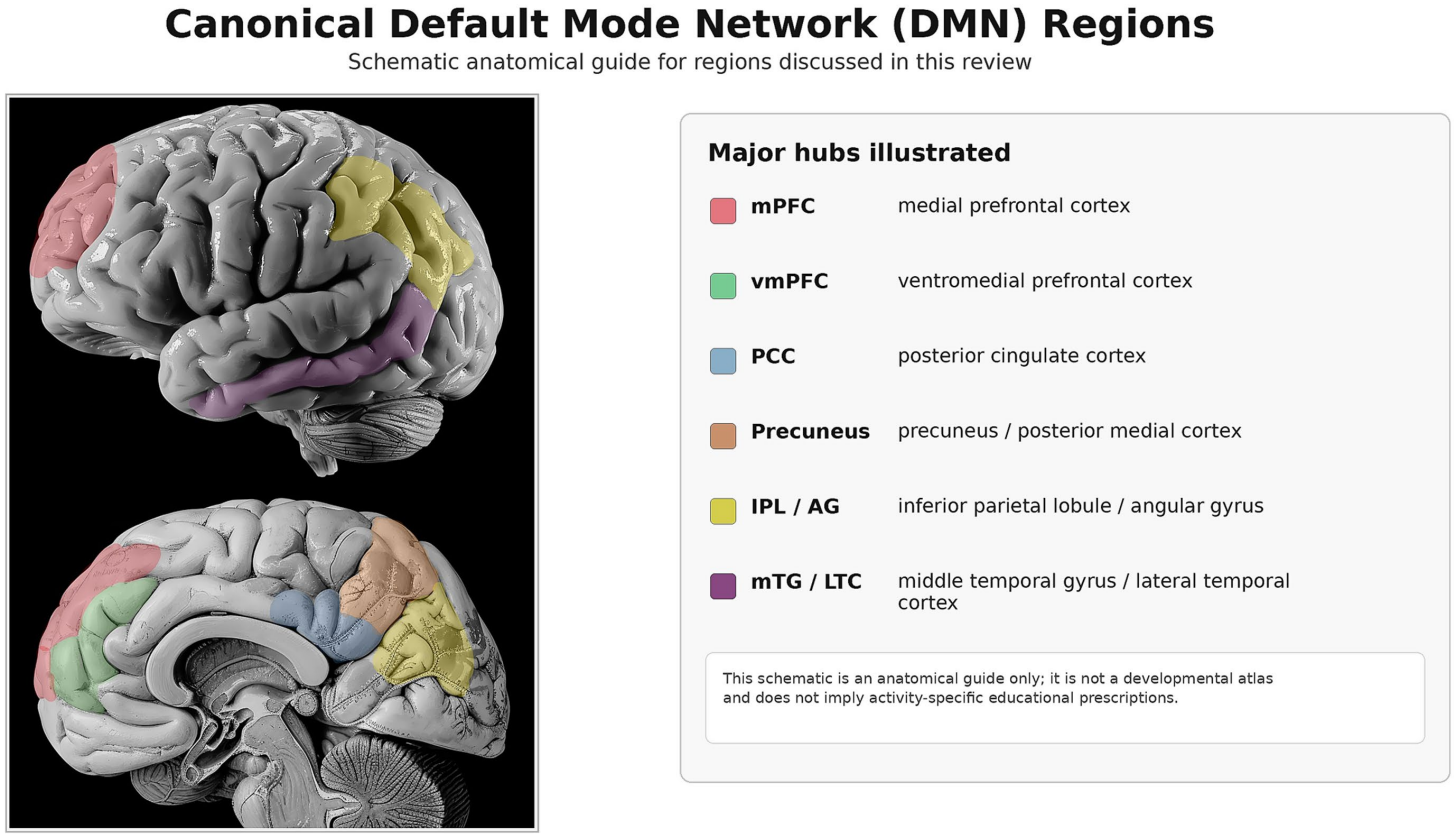
Schematic anatomical guide to canonical default mode network regions discussed in this review. Adapted from Azarias et al. (2025), Figure 2, “The Journey of the Default Mode Network: Development, Function, and Impact on Mental Health,” Biology, 14, 395, under the Creative Commons Attribution 4.0 International License (CC BY 4.0). The original figure was cropped, resized, and simplified by removing extended explanatory text and condensing labels.

The DMN is typically described as a distributed set of midline and lateral parietal/temporal regions—including medial prefrontal cortex (mPFC), posterior cingulate cortex/precuneus (PCC/Prec), angular gyrus (AG), and medial temporal lobe structures—whose activity often decreases during demanding externally oriented tasks and increases during internally oriented cognition (Raichle et al., 2001; Buckner et al., 2008; Raichle, 2015). Figure 2 provides a schematic anatomical guide to the canonical DMN regions discussed in this review. Contemporary views emphasize that the DMN is not a unitary “rest network,” but a heterogeneous system with subsystems and connector hubs that can couple flexibly with memory, language, and control systems depending on context (Andrews-Hanna et al., 2010; Smallwood et al., 2021). Menon (2023) synthesizes this literature to propose that DMN dynamics help integrate and broadcast memory, language, and semantic representations in ways that can sustain a coherent internal narrative, supporting subjective continuity and aspects of selfhood.

A foundational reason DMN science matters for learning and development is that cognition appears organized around mode switching: the brain alternates between externally driven processing (attention to stimuli and task execution) and internally driven processing (retrieval, integration, simulation, and interpretation). From this perspective, “internal” modes are not merely lapses in engagement; they can be part of how experiences are consolidated, interpreted, and connected to prior knowledge and personal meaning. Immordino-Yang (2016) highlights a constraint relevant to this broader developmental picture: it can be difficult to simultaneously devote full attention to completing a current task and to reflect on the broader meaning and implications of that task. This cautions against equating learning with uninterrupted external task engagement and invites attention to the timing and conditions under which constructive reflection is most likely to occur. This internal–external distinction is a heuristic rather than a strict dichotomy (Smallwood et al., 2021; Menon, 2023): DMN regions can support externally oriented tasks when they require narrative, semantic, or social integration (Lerner et al., 2011; Fernandino and Binder, 2024; Spreng et al., 2009). Semantic cognition depends on distributed heteromodal systems that transform sensory and linguistic input into conceptual meaning; this provides an important bridge between DMN accounts of internal meaning-making and broader neurocognitive models of semantic representation (Binder and Desai, 2011; Lambon Ralph et al., 2017; Fernandino and Binder, 2024). This translational interpretation is consistent with peer-reviewed perspective work arguing that rest and internally oriented processing should not be treated as idleness, but as developmentally meaningful conditions for reflection, social–emotional meaning-making, and educational practice (Immordino-Yang et al., 2012; Immordino-Yang, 2016). Accordingly, the educational question is not “DMN on versus off” but whether contexts allow cycles of engagement and integration (Immordino-Yang, 2016; Menon, 2023).

Importantly, DMN function is not reducible to “distraction,” even though the network is often deactivated during goal-oriented tasks and activated during mind-wandering and daydreaming. Meta-analyses and theoretical reviews implicate DMN regions in multiple constructive processes central to development, including autobiographical memory, prospection, semantic integration, social cognition, and narrative comprehension (Spreng et al., 2009; Schacter et al., 2007; Yeshurun et al., 2021). These processes support widely recognized developmental achievements—constructing coherent accounts of experience, understanding others’ intentions, imagining diverse possibilities and coming up with creative solutions, and building increasingly stable self-representations—without implying that such abilities are uniquely or exclusively DMN-based, or that more DMN is inherently better in all contexts.

Language and meaning-making provide a useful case for specifying what “DMN involvement” does—and does not—imply for learning and development. DMN hubs are most consistently recruited when comprehension requires integrating linguistic input into a coherent, high-level situation model across extended time. Work on hierarchical temporal processing shows that regions overlapping the DMN—especially posterior midline cortex (PCC/precuneus) and inferior parietal areas (including angular gyrus)—exhibit long temporal receptive windows, tracking information accumulated over tens of seconds to minutes; their response reliability drops when narratives are temporally scrambled, consistent with a role in discourse-level integration rather than sentence-by-sentence processing (Lerner et al., 2011; Hasson et al., 2015). Within this framework, the angular gyrus (a canonical DMN node) is repeatedly implicated in multimodal semantic integration and contextual meaning construction, complementing widely distributed semantic representational systems and semantic control mechanisms (Binder and Desai, 2011; Lambon Ralph et al., 2017; Seghier, 2013). Recent work further clarifies how DMN organization may contribute to semantic cognition at the network level (Fernandino and Binder, 2024). Converging naturalistic neuroimaging studies also suggest that shared understanding of the same story is accompanied by across-person alignment in midline DMN regions (intersubject correlation) and by speaker–listener coupling, consistent with the idea that communication relies on partially shared high-level situation models instantiated in transmodal hubs (Hasson et al., 2012; Stephens et al., 2010; Yeshurun et al., 2021). Developmentally, the educational relevance is straightforward: growth in discourse comprehension, narrative coherence, and conceptual learning depends heavily on integrating language with memory, prior knowledge, and social inference—domains that are strongly compatible with, though not reducible to, DMN-linked transmodal integration.

The DMN is also implicated in event segmentation and event memory, which is critical for learning and various domains of development, not because it “stores memories” per se, but because key DMN nodes appear to represent and update high-level situation models over long temporal windows. During naturalistic comprehension, posterior midline DMN hubs (including PCC/precuneus) show transient responses at moments later judged to be event boundaries, consistent with the idea that boundaries trigger updating of the currently active situation model (Speer et al., 2007). Extending this, data-driven “event-structure” modeling of continuous narratives indicates a cortical hierarchy in which short, fast-changing event representations appear in sensory regions, whereas longer, more abstract event representations emerge in high-order areas that overlap canonical DMN territory—particularly angular gyrus and posterior medial cortex—interpreted as supporting multimodal situation models (Baldassano et al., 2017). Critically, high-level event boundaries in these DMN-overlapping regions are coupled to increased hippocampal activity, and boundary-linked hippocampal responses predict subsequent reinstatement and later free recall, suggesting a mechanism by which event-model transitions in cortex and episodic encoding in the hippocampus become coordinated (Baldassano et al., 2017). In this framing, the DMN’s contribution is most precisely located in maintaining and revising integrative event models (context, goals, and narrative state), thereby providing structured cortical “units” that can be bound into long-term episodic memory traces through hippocampal–cortical interaction (Ranganath and Ritchey, 2012).

Rest and offline processing provide another domain in which DMN dynamics intersect with learning and development through memory consolidation mechanisms. The DMN is reliably engaged during wakeful rest, a state in which cognition is often internally oriented and recent experiences can be spontaneously reactivated and reorganized. Human neuroimaging studies show that patterns characteristic of prior encoding persist into immediate post-encoding rest and that the degree of such persistence relates to later memory performance, consistent with offline reactivation supporting consolidation (Tambini and Davachi, 2013). Related work demonstrates that post-task resting-state hippocampal–cortical coupling can be selectively enhanced following successful encoding and can predict individual differences in later associative memory, supporting the broader claim that rest is an active period for hippocampal–neocortical coordination (Tambini et al., 2010). Where the DMN enters most clearly is at the network level: default-network regions are among the cortical systems that are active “by default” during rest, and evidence indicates that increased functional coupling among default-network nodes during post-encoding rest can track subsequent memory outcomes—illustrated, for example, by findings that post-encoding connectivity among MPFC–TPJ and other default-network regions relates to memory for newly learned social information (Meyer et al., 2019). Mechanistically, this is consistent with accounts in which hippocampal reactivation during rest is integrated into distributed cortical representations supported by transmodal association networks; notably, resting-state work also suggests that medial temporal lobe pathways (e.g., parahippocampal gyrus) provide a major route linking hippocampal memory systems to cortical DMN hubs (Ward et al., 2014). Taken together, DMN relevance here is not that “rest equals DMN,” but that DMN-dominant rest states may provide a neural context in which reactivated memory traces can be integrated, stabilized, and—over time—abstracted into more generalizable knowledge structures.

Finally, the DMN is frequently discussed in relation to imagination and creative cognition because it supports self-generated mental simulation and associative recombination of memory content—processes that can generate candidate ideas in the absence of immediate external constraints. Mind wandering, a common form of spontaneous self-generated thought, is behaviorally associated with creative “incubation,” such that undemanding breaks that permit off-task thought can improve subsequent performance on divergent-thinking problems (Baird et al., 2012). Resting-state connectivity evidence also links default-network organization to individual differences in creative cognition (Beaty et al., 2014). Importantly, contemporary network accounts do not treat creativity as a pure DMN function. Instead, they propose that creative cognition depends on dynamic interaction between internally generative processes (often linked to DMN activity) and control/selection processes (linked to executive-control systems) that evaluate, constrain, and refine candidate ideas. Consistent with this, fMRI studies show that creative idea production can involve functional coupling between hubs of the default network (e.g., PCC/precuneus and inferior parietal regions) and executive-control regions (e.g., dorsolateral prefrontal cortex), indicating cooperative dynamics rather than strict antagonism during creative thought (Beaty et al., 2015). Creative problem solving often cycles between a generative mode (DMN-biased, producing candidate associations by recombining memory and concepts) and an evaluative/refinement mode (executive-control-biased, maintaining goals and constraints and elaborating the most promising candidates). Functional coupling likely indexes moments when a newly generated association is brought under goal-directed scrutiny and shaped into a viable idea. From a developmental perspective, the key implication is that imagination and creativity can be understood as emerging capacities for goal-directed, self-generated thought—supported by maturing coordination between DMN-based simulation/recombination and control systems that stabilize and shape the products of that simulation into usable plans, explanations, or novel solutions.

### 3.2 Developmental trajectory of the DMN from infancy through preschool

Evidence indicates that elements of DMN emerge early but undergo major reorganization across the first years of life (Gao et al., 2009; Gao et al., 2015b). Rather than a single linear strengthening, early DMN development is best conceptualized as a multi-dimensional transformation that includes (a) the appearance of recognizable DMN-related nodes, (b) age-related changes in the spatial extent and stability of those nodes, (c) progressive strengthening of long-range (especially anterior–posterior) coupling (e.g., mPFC–PCC/precuneus), and (d) gradual differentiation of DMN subsystems alongside changing cooperative/competitive relationships with other large-scale networks supporting externally oriented attention and executive control (Cao et al., 2017; Gao et al., 2013; Gao et al., 2015b; Xiao et al., 2016). Because the DMN is a transmodal association network whose canonical hubs lie in heteromodal cortex, its maturation is closely tied to prolonged neurodevelopmental processes (e.g., synaptic refinement, myelination of long-range association pathways, and consolidation of distributed cortical hierarchies) that proceed on a different timetable than primary sensory systems (Buckner et al., 2008; Cao et al., 2017; Huttenlocher and Dabholkar, 1997; Margulies et al., 2016). In this framing, immature DMN organization should not be equated with deficit; it can reflect a normative developmental stage in which the brain is still constructing infrastructure for flexible coordination between internally and externally oriented modes of processing (Cao et al., 2017; Fransson et al., 2011; Gao et al., 2009).

Neonatal and early-infant fMRI studies indicate that rudimentary resting-state organization emerges early, especially in sensory, sensorimotor, auditory, and visual systems. However, evidence for a complete DMN precursor in the neonatal period is mixed: Fransson et al. (2007) identified several infant RSNs but failed to detect a direct adult-equivalent default-mode network, and larger dHCP work similarly found adult-like topography in primary RSNs but not a complete DMN precursor at term-equivalent age. More direct longitudinal evidence suggests that DMN-related nodes and partial configurations become increasingly integrated across the first two years (Fransson et al., 2007; Gao et al., 2009). Research across the third trimester and into term-equivalent age therefore supports a primary-to-association-network developmental sequence, in which early RSNs are detectable but higher-order association networks remain fragmented, state-sensitive, and vulnerable to prematurity or perinatal adversity (Doria et al., 2010; Smyser et al., 2010; Eyre et al., 2021). Clinical and high-risk studies provide additional boundary conditions rather than direct evidence of normative development: dHCP analyses of full-term, preterm term-equivalent, and preterm-before-term-equivalent infants suggest that default/frontoparietal circuitry and reciprocal DMN–attention relationships are age-sensitive and vulnerable to prematurity, while perinatal brain-injury work shows that disruption to early functional networks can predict later motor outcomes (Hu, H. et al., 2022; Linke et al., 2018). These findings are treated here as contextual constraints rather than primary evidence for typical DMN maturation. In other words, early development begins with recognizable network motifs, but these motifs are sparse, state-sensitive, and still being tuned and integrated with maturing structural pathways.

A key interpretive point is that adult-like topography does not imply adult-like function (Cao et al., 2017; Menon, 2023). By toddlerhood, DMN-like regions can often be mapped in ways that resemble the broad spatial layout of the adult network (Gao et al., 2009; Gao et al., 2015b; Kardan et al., 2022), yet functional meaning depends on how subsystems are organized (Andrews-Hanna et al., 2010; Xiao et al., 2016) and how the DMN dynamically couples with salience, attention, and control systems during cognition in naturalistic contexts (Smallwood et al., 2021; Menon, 2023). Developmentally, the same anatomical nodes may participate in different computations, operate on different timescales, or couple with different partners than they do in mature brains, making it essential to track not only detectability but also internal coherence, subsystem structure, and between-network organization over time (Cao et al., 2017; Fransson et al., 2011; Xiao et al., 2016; Yin et al., 2025).

From a within-network maturation perspective, longitudinal evidence across the first postnatal year suggests a relatively ordered sequence in which primary sensory systems stabilize earlier and higher-order systems (including the DMN) mature later. In one influential longitudinal study with repeated scans across infancy, primary sensorimotor, auditory, and visual networks resembled adult-like topologies soon after birth and changed modestly, whereas attention, default-mode, and frontoparietal/executive systems were less adult-like at birth and showed age-dependent increases in connectivity, with some DMN components approaching more adult-like topology by the end of the first year (Gao et al., 2015a; Gao et al., 2015b). Here, “adult-like topology” is best understood as increasing detectability of canonical nodes and stronger coherence among them, rather than mature functional specialization. Related connectomics syntheses emphasize that early development involves increasing long-range integration alongside increasing segregation (specialization) of systems, with hub organization shifting from primary sensory/motor regions toward heteromodal association cortex (Cao et al., 2017). Consistent with this, infant hub organization appears more concentrated in primary sensory and motor regions than in heteromodal association areas, suggesting that adult-like prominence of transmodal hubs (including DMN hubs) is not fully established in early infancy (Fransson et al., 2011).

From an inter-network coordination perspective, a developmentally salient milestone is the gradual emergence of coordinated competition between systems supporting internal versus external attention. In early infancy, default and dorsal attention systems begin as relatively isolated regions and become more internally synchronized over the first year; anticorrelated interactions between default and dorsal attention networks—often interpreted as an index of increasingly organized switching between internally oriented and externally oriented modes—are typically weak or absent at birth but become more evident around one year and strengthen further by age two (Gao et al., 2013). This implies that the neural infrastructure for toggling between “outer” and “inner” modes is itself a developmental achievement; variability in attention and transitions is expected in infants and young toddlers as these large-scale relationships are still stabilizing.

Methodological considerations are especially important when interpreting these developmental patterns. First, “anticorrelation” estimates can be sensitive to denoising choices, most notably global signal regression (GSR), which removes variance shared across much of the brain and can shift correlation distributions toward zero—thereby increasing the prevalence and magnitude of negative correlations (Murphy et al., 2009; Saad et al., 2012). At the same time, GSR can reduce widespread artifacts related to head motion and physiological fluctuations—factors that differ systematically by age—so developmental conclusions about emerging network competition should be tested for robustness across plausible preprocessing pipelines rather than relying on a single analytic choice (Ciric et al., 2017; Murphy and Fox, 2017). Second, acquisition state is a major issue in infancy: resting-state fMRI is often obtained during natural sleep to reduce motion, whereas most adult resting-state data are collected in wakefulness. Sleep-state connectivity can differ from awake connectivity, including reduced DMN functional connectivity during sleep, and direct comparisons of infants scanned during natural sleep versus awake movie-watching confirm state-dependent differences in functional connectivity patterns (Mitra et al., 2017; Yates et al., 2023). Together, these points motivate cautious interpretation of infant–adult differences and emphasize that “resting-state” in infancy is not a single physiological condition.

Recent trajectory and “brain chart” approaches extend earlier infancy and preschool findings by quantifying normative within and between-network developmental curves across birth to 6 years in large samples. A large-scale aggregation of 1,091 resting-state scans delineated developmental charts of functional connectivity within and between canonical networks, explicitly modeling and harmonizing differences between asleep and awake acquisitions (Yin et al., 2025). Intranetwork curves suggested that the default network shows an early rapid maturation pattern with a peak/transition point around 16 months, whereas dorsal attention shows a more delayed and protracted increase and control networks continue to rise across early childhood (Yin et al., 2025). Importantly for claims about “competition,” internetwork curves indicated that network relationships are pair-specific: some network pairs remain consistently positive, others consistently negative, and a substantial subset shows transitional trajectories that shift across positive, near-zero, and negative regimes over development (Yin et al., 2025). For default-network interactions specifically, default–control coupling remained positive across 0–6 years but decreased steadily with age, whereas default–dorsal attention coupling became progressively more negative through early childhood and stabilized later; moreover, not all adult-like antagonisms were present early, with ventral attention–default anticorrelation emerging around 2 years as a transition rather than being established at birth (Yin et al., 2025). The same work further linked deviations from normative curves to early cognitive measures, supporting the idea that variability in the maturation of default, attention, and control-related connectivity patterns is meaningfully related to behavioral development (Yin et al., 2025). Although these charts require replication and continued attention to harmonization and state inference, they sharpen empirical claims about timing, variability, and candidate milestones in early DMN-related organization. Related recent work extends trajectory mapping into the prenatal/newborn period and highlights early state-sensitive network organization (Scheinost et al., 2024; Derman and Ferradal, 2025).

Additional dHCP and BCP studies further refine this developmental picture. Dynamic functional-connectivity analyses of term and preterm neonates suggest that neonatal brain states are already organized in functionally meaningful ways and relate to early developmental outcomes, while still showing maturational and prematurity-related differences (França et al., 2024). The BCP protocol provides methodological background for several infant-connectome analyses (Howell et al., 2019), and BCP-based work also indicates that infancy is characterized not only by strengthening of selected connections but by broader changes in individual functional-connectome fingerprints, cortical parcellation, and regional network efficiency during the first two years of life (Hu D. et al., 2022; Wang et al., 2023; Jiang et al., 2023). These studies should not be overread as direct evidence for classroom practices; rather, they strengthen the developmental premise that early DMN-related organization is embedded in rapidly changing whole-brain network architecture.

Beyond temporal coupling, infancy and toddlerhood are also characterized by pronounced spatial reorganization—i.e., how consistently network patterns occupy their “adult-like” locations and how sharply network boundaries are expressed. Longitudinal analyses from birth to 6 months show systematic age-related changes in spatial characteristics of networks (e.g., alignment to group-level maps, spatial extent, within-network voxel contribution), consistent with a consolidation process in which network configurations become more consistent and less spatially variable over time (Seraji et al., 2025). In slightly older toddlers and young children, related work suggests that DMN development can manifest as decreasing spatial variability and increasing homogeneity rather than uniform strengthening of all edges: in toddlers scanned during natural sleep, reduced variability (increased homogeneity) in a posterior DMN component related to age, and greater homogeneity in higher-order networks—including posterior DMN—related to more advanced developmental skills (Chen et al., 2021).

A final complementary line of evidence comes from individual-differences and reliability-focused analyses in the second year of life. Using Baby Connectome Project data from 8-to-26-month-olds, Kardan et al. (2022) showed that infant/toddler functional connectomes demonstrate measurable within-session reliability and can be distinctive enough for “connectome fingerprinting,” and that multivariate patterns of functional connectivity predict age with meaningful accuracy. Notably, age-predictive information was not dominated by primary sensory networks; connections involving attention systems and higher-order networks contributed strongly, consistent with the broader view that the second year of life is a period of notable maturation in systems relevant to flexible attention, emerging self–other representations, and memory integration (Alcauter et al., 2014; Bulgarelli et al., 2019; Kardan et al., 2022; Liu et al., 2021). This provides quantitative evidence that higher-order network organization changes rapidly enough in toddlerhood to serve as a developmental marker, even while functional specialization remains immature relative to later childhood.

Preschool findings provide additional evidence that DMN development involves subsystem differentiation rather than uniform strengthening. Building on the adult literature that distinguishes a dorsomedial prefrontal (dMPFC) subsystem often linked to mentalizing/self-relevant social cognition and a medial temporal lobe (MTL) subsystem linked to memory and scene construction, preschool data suggest partially distinct developmental trajectories across ages 3–5: age-related changes were more reliably expressed in the MTL subsystem than the dMPFC subsystem, cross-subsystem coupling increased by age 5 (particularly in left-hemisphere components), and lateralization shifted from stronger right-hemisphere dominance at age 3 toward more bilateral organization by age 5 (Xiao et al., 2016). More broadly, the preschool period underscores that DMN development unfolds within a rapidly changing network ecology: even when DMN nodes are identifiable, their relationships with attention, salience, and control systems continue to differ from later childhood and adulthood, and stable adult-like antagonism is a later achievement rather than an early default.

In sum, the infancy-to-preschool trajectory of the DMN is best characterized not as the early presence of a mature network, but as early RSN organization and partial DMN-related motifs followed by prolonged default-network refinement across multiple dimensions: increasing detectability and coherence of canonical nodes, strengthening of long-range integration, progressive stabilization of inter-network competition/cooperation patterns, and spatial sharpening and subsystem differentiation through toddlerhood and preschool. State (sleep vs. wake engagement), analytic choices, and individual variability are central determinants of what can be inferred about early DMN organization. From a developmental standpoint, the most defensible conclusion is not that young children should be pushed to “activate the DMN,” but that early development naturally entails alternating and gradually coordinating internally oriented integration and externally oriented engagement. Educationally, this shifts the question from how to accelerate a neural system to how everyday environments can protect the rhythms, relationships, and meaning-making opportunities through which such coordination is gradually supported.

### 3.3 DMN-informed educational principles for early childhood practice

The aim of this section is not to create an extensive list of DMN-based classroom activities or to suggest that children should be “trained” to engage the DMN. Instead, this section translates the preceding neuroscientific and developmental synthesis into four conservative design principles for early childhood settings. These principles concern the organization of everyday learning conditions: rhythms of engagement and integration, narrative meaning-making, emotionally secure co-regulation, and constructive solitude within a socially rich ecology. They are DMN-informed only in the limited sense that they align with cognitive functions and developmental constraints highlighted by DMN research; they are not claims of direct neural causality.

#### 3.3.1 Age-general principles

##### 3.3.1.1 Principle 1: protect engage–pause–integrate rhythms rather than organizing the day as continuous demand

A core constraint emerging from DMN research is that reflective integration and instrumental task focus are difficult to optimize at the same moment. When external demands are high, internally oriented integrative processing (e.g., memory replay, semantic integration, simulation, and interpretation) is less likely to unfold; conversely, when external demands ease, internally oriented processes can re-emerge and help consolidate and “absorb” what has been experienced (Immordino-Yang, 2016; Menon, 2023). For early development, this supports a familiar but often eroded logic of daily rhythms: alternating externally guided, rule-bound, stimulus-rich group activity with periods that allow children to downshift into quieter, self-directed, playful, or restful states. Such pauses are not “empty time”; they plausibly function as windows for monitoring, reorganizing, and integrating recent experience into memory and emerging understanding, consistent with experimental, review, and meta-analytic evidence that post-encoding wakeful rest can support memory consolidation, while also showing that rest effects depend on task conditions, memory materials, and the nature of post-encoding activity (Tambini and Davachi, 2013; Wamsley, 2022; Weng et al., 2025). A DMN lens therefore does not introduce new content so much as it offers convergent mechanistic support for protecting the temporal architecture of early learning—pauses, calm transitions, unstructured play, and protected rest—so that engagement is repeatedly followed by opportunities for integration rather than being treated as a continuously optimized performance stream (Luo et al., 2024).

##### 3.3.1.2 Principle 2: treat narrative and meaning-making as developmental infrastructure

Integrative accounts emphasize that DMN dynamics support the construction of internal narratives and the binding of experience into coherent meaning across time (Menon, 2023). Developmental evidence similarly indicates that autobiographical memory and the narrative self-emerge gradually across the preschool years through the interplay of language, narrative practices, temporal understanding, and developing concepts of self and other (Nelson and Fivush, 2004; Fivush, 2011). From this perspective, narrative should be treated as learning infrastructure: children do not merely accumulate facts; they learn by building situation models (“what happened, why it happened, how it felt, what it means, what comes next”) that connect new information to prior knowledge and personal experience. This framing helps clarify why early childhood practices such as storytelling, shared book reading, reenactment in socio-dramatic play, and reflective conversations about everyday events are not simply “language enrichment,” but developmentally appropriate formats for integration—supporting the growth of coherent memory, conceptual understanding, and self-relevant meaning over time (Hasson et al., 2012; Yeshurun et al., 2021).

Consistent with this framing, meta-analytic and intervention evidence indicates that dialogic book reading and elaborative reminiscing support young children’s language, narrative, and autobiographical memory development (Mol et al., 2008; Reese and Newcombe, 2007; Pillinger and Vardy, 2022). Narrative-rich practices extend beyond dialogic picture-book reading alone. In preschool settings, children are generally invited to retell familiar stories, dramatize picture books, create alternative endings, dictate or draw their own stories, engage in story-making with peers, and enact roles in socio-dramatic play for the development of oral language, emergent literacy, and social competence (Lever and Sénéchal, 2011; Nicolopoulou et al., 2015). The value of these activities lies in repeatedly inviting children to hold a story world in mind across time—characters, goals, causal changes, emotions, and perspectives—and to connect that world with prior knowledge and personal experience. In neuroscience research, these operations are strongly aligned with canonical DMN and DMN-overlapping regions: posterior medial cortex/PCC–precuneus and angular gyrus for extended situation-model integration; medial temporal and lateral/anterior temporal regions for episodic/autobiographical and semantic information; and mPFC/TPJ for self–other and mental-state reasoning (Spreng et al., 2009; Lerner et al., 2011; Binder and Desai, 2011; Seghier, 2013; Baldassano et al., 2017; Yeshurun et al., 2021; Menon, 2023). Thus, teacher prompts such as “What happened first?,” “Why did she do that?,” “How did he feel?,” “Have you ever felt that way?,” and “What might happen next?” can be described more conservatively as scaffolds for temporal sequencing, causal explanation, emotion language, perspective coordination, and autobiographical coherence, capacities supported behaviorally by elaborative reminiscing and narrative-interaction research (Nelson and Fivush, 2004; Fivush et al., 2006; Reese and Newcombe, 2007; Fivush, 2011).

##### 3.3.1.3 Principle 3: protect emotionally secure, socially meaningful contexts that make reflection possible

Across DMN-oriented accounts, reflective integration is intertwined with social emotion, personal memory, future-oriented thinking, and conceptual understanding (Immordino-Yang, 2016; Menon, 2023). For young children, reflective meaning-making is therefore unlikely to be separable from felt safety, responsive relationships, and co-regulation. Emotionally secure contexts are not an “extra” condition; they are part of the cognitive ecology that enables children to revisit experience, explore interpretations, and tolerate ambiguity and curiosity without excessive threat-monitoring. When a child is preoccupied with anxiety, separation distress, social threat, or fear of failure, cognitive resources are more likely to be recruited for monitoring threat and managing arousal than for flexible exploration, narrative revisiting, or inward integration of experience (Shonkoff et al., 2012; Vogel and Schwabe, 2016). In such states, children may be physically present during an activity but less able to take in external input deeply enough for later internalization and meaning-making. Emotional security therefore functions as a precondition for the engage–pause–integrate rhythm: children need enough felt safety to attend outwardly, and then enough calm to turn inward and reorganize what has been experienced. The implication is not that classrooms should eliminate all challenge or frustration. Rather, challenge should be embedded in predictable routines, warm teacher–child relationships, and co-regulatory support so that arousal remains within a range compatible with curiosity, reflection, and memory integration (Gunnar and Quevedo, 2007; Shonkoff et al., 2012; Vogel and Schwabe, 2016; Immordino-Yang, 2016).

##### 3.3.1.4 Principle 4: normalize constructive solitude and self-directed activity within a social learning ecology

Early childhood settings sometimes treat being alone as a problem to be corrected, assuming that social development requires continuous group participation. However, developmental research distinguishes among forms of solitude: some solitary behavior reflects anxiety or social fear (reticence), but other forms (solitary-passive constructive/exploratory play, solitary-active functional/dramatic play) can be stable and not inherently maladaptive (Coplan et al., 1994; Rubin et al., 2009). A DMN lens offers an additional interpretive frame: development also requires opportunities for internally oriented, self-generated thought—periods in which children are not responding to immediate external demands but are instead consolidating, simulating, and organizing experience in their own way. In early childhood classrooms, such internally oriented time may coincide with quiet solitary play, absorbed imaginative activity, or private/inner speech as children begin to use language to guide their own attention and action (Alderson-Day and Fernyhough, 2015). The educational implication is not to romanticize isolation or to ignore social-withdrawal risk, but to refine adult interpretation: teachers can differentiate a child who is distressed, excluded, or avoidant from a child who is calmly absorbed in self-directed exploration. Protecting appropriate solitude within a socially supportive classroom may therefore complement social learning, by allowing children to consolidate experience, rehearse narratives, and generate ideas without constant external direction (Rubin et al., 2009; Smallwood et al., 2021).

At the same time, a DMN-informed view should not be read as an argument for reducing social interaction. DMN and DMN-overlapping regions are consistently implicated not only in self-generated thought but also in mentalizing, perspective taking, and self–other modeling, with recurrent involvement of medial prefrontal cortex, posterior temporoparietal junction, precuneus/posterior medial cortex, and temporal-lobe regions (Spreng et al., 2009; Mars et al., 2012; Schurz et al., 2014; Yeshurun et al., 2021; Menon, 2023). Therefore, protecting solitary absorption should not mean reducing opportunities for peer conversation, collaborative play, or teacher–child dialogue. Socio-dramatic play is especially relevant because it brings self-generated narrative and social coordination together. When children negotiate roles, sustain a pretend scenario, repair misunderstandings, and talk about “what is happening” inside the play frame, they must hold a story world in mind while tracking roles, intentions, beliefs, emotions, and expectations. Behaviorally, such play affords practice in narrative coherence, self-regulatory language, metacommunication, and social perspective-taking, although evidence should not be overstated as proving unique causal effects of pretend play on development (Elias and Berk, 2002; Lillard et al., 2013; Nicolopoulou et al., 2015). In a construct-mapping sense, socio-dramatic play can therefore be described as DMN-consistent: it supports the behavioral coordination of narrative, memory, role, and perspective, without implying that pretend play directly activates or trains the DMN.

#### 3.3.2 Age-specific considerations across early developmental windows

The gradual refinement of the DMN is situated within broader neurodevelopmental processes of synaptic overproduction, pruning, and myelination. Early synaptogenesis provides a highly plastic substrate for experience-dependent learning, but efficient large-scale cognition requires subsequent refinement of redundant or weakly stabilized connections and strengthening of frequently coordinated pathways. This pruning/refinement process should not be interpreted simply as “loss”; rather, it is part of the sculpting of more efficient, specialized, and integrated network organization. Because canonical DMN hubs are located largely in heteromodal association cortices, which mature more protractedly than primary sensory systems, DMN development is plausibly constrained by prolonged synaptic refinement and the maturation of long-range association pathways (Huttenlocher and Dabholkar, 1997; Petanjek et al., 2011; Cao et al., 2017). From an educational standpoint, this reinforces the need for developmental humility: early childhood is not a period in which adult-like internal mentation can be simply accelerated, but a period in which repeated, emotionally secure, meaningful experiences may gradually contribute to the functional organization of memory, language, social understanding, and self-related thought.

In this context, age bands here are used as pragmatic anchors rather than discrete developmental stages. The considerations below integrate (a) evidence that early RSN organization, DMN-related nodes, or partial configurations become more coherent gradually with long-range integration, hub organization, and coordination with attention/control systems remaining strongly state-sensitive in the first years of life (Fransson et al., 2007; Gao et al., 2009; Gao et al., 2013; Gao et al., 2015b), with (b) well-established developmental tasks and constraints in early childhood (e.g., sleep–wake organization in infancy; rapid growth in symbolic representation and language in toddlerhood). Accordingly, the implications are framed as conservative context-level considerations—conditions that plausibly support cycles of externally oriented engagement followed by internally oriented integration—rather than as neural prescriptions. Table 1 summarizes these age-banded considerations. To avoid implying discrete stages, the text below highlights the dominant developmental constraint in each window, while Table 1 provides illustrative practice-aligned conditions.

**Table 1:** Illustrative alignment between early RSN/DMN-related network development and common ECE practice.

Age range	Key patterns in DMN/network development and broader developmental constraints (brief)	Context-level ECE conditions aligned with the developmental interpretation (illustrative; non-prescriptive)
Birth–18 months	DMN-like fragments detectable, but long-range integration/hub organization and mode switching are immature; strong sensitivity to behavioral state (sleep vs. wake). Large individual variability in sleep–wake rhythms; development is strongly scaffolded by caregiver co-regulation; early maturation prioritizes sensory/sensorimotor systems relative to higher-order networks.	Align routines to infant rhythms (sleep/feeding/alertness); emphasize responsive co-regulation and calm transitions; provide rich sensorimotor exploration (self-paced movement, object play) interleaved with brief low-stimulation intervals; protect adequate sleep opportunities and minimize overstimulation; avoid sustained attention demands as an expectation; provide vivid sensory and sensorimotor experiences—touch, movement, object exploration, rhythm, caregiver voice, and shared gaze—as early experiential material for later memory, language, and meaning-making; protect quiet intervals after stimulation rather than filling every pause with additional input.
18–36 months	Rapid reorganization of higher-order networks; emerging default–attention competition and high variability in switching/attention. Rapid growth in language, symbolic representation, memory integration, and early self-concept; autonomy and self-regulation are emerging and context-dependent.	Use predictable transition cues (advance warnings, routines, songs); alternate brief adult-guided engagement with extended child-initiated free play and quiet downtime; embed frequent revisiting of experience (retell, reenact, draw, quiet observation); interpret private speech as a potentially adaptive self-regulatory tool while scaffolding participation in shared routines and peer interaction; support simple oscillations between social participation and self-directed absorption: brief peer or caregiver interaction followed by time to reenact, repeat, name, draw, or privately narrate the experience.
3–5 years	DMN is identifiable; subsystem differentiation continues; coordination with control networks improves but remains effortful/context-dependent. Longer pretend-play scripts and multi-episode narratives become more feasible; executive control is developing but still fragile under high demand.	Scale up integration demands (longer narratives, projects, planning–revision cycles): pair goal-based tasks with explicit integration windows (reflection, reenactment, discussion, or play); support both collaboration and quiet absorbed activity; avoid continuous rapid task switching and uninterrupted performance demands; use socio-dramatic play, collaborative story-making, construction play, movement, drawing, and reflective conversation as complementary contexts in which children gather vivid sensory–motor and social experiences, then reorganize them through narrative, imagination, and quiet self-directed thought.

Age bands are heuristic anchors. The third column highlights context-level conditions commonly present in high-quality ECE settings that plausibly support cycles of engagement and integration, without implying DMN-specific causal effects. These examples should be read as developmentally grounded design considerations, not as age-based prescriptions or neural intervention targets.

##### 3.3.2.1 Birth–18 months: integration in short cycles, with high state sensitivity

DMN-like regions can be detected early, but long-range integration, hub organization, and coordination with attention systems are still forming and are strongly modulated by behavioral state in infancy (Fransson et al., 2007; Gao et al., 2009; Gao et al., 2013; Gao et al., 2015b). This period is also characterized by substantial individual variability in sleep duration and sleep–wake organization across the first year, underscoring that “rhythm” constraints must be interpreted through the infant’s biological and family rhythms rather than fixed schedules (Iglowstein et al., 2003). Educationally, the most defensible priorities are therefore foundational: protecting sufficient sleep, feeding, and nutrition that support rapid neurodevelopment (Cusick and Georgieff, 2016), and maintaining emotionally secure, responsive co-regulation during transitions. Because primary sensory and sensorimotor systems mature earlier than higher-order networks (Gao et al., 2015a; Cao et al., 2017), early settings can emphasize rich sensorimotor exploration (repetitive, self-paced movement and object play) interleaved with brief low-demand intervals (quiet looking, cuddling, unstructured gaze) that allow endogenous activity to unfold.

##### 3.3.2.2 18–36 months: emerging competition and increasing variability in switching

Across the second and third years, within-network synchronization strengthens and inter-network relationships become more organized, including the emergence and strengthening of default–dorsal attention competition that is often interpreted as increasingly organized switching between internally and externally oriented modes (Gao et al., 2013; Yin et al., 2025). These network-level changes coincide with rapid growth in symbolic representation and language, and with the beginnings of narrative self-organization (Nelson and Fivush, 2004; Fivush, 2011). Consequently, rhythm design can become more intentional: predictable cues and emotionally supportive transitions (songs, routines, and advance warnings) can help toddlers shift between adult-guided episodes of focused engagement and extended blocks of child-initiated free play and quiet downtime. In this window, overt private speech is developmentally salient and educationally relevant: self-directed talk during play and problem solving is implicated in emerging self-regulation and is theorized to become internalized as inner speech over time (Whedon et al., 2021; Alderson-Day and Fernyhough, 2015). While direct evidence connecting private speech to DMN dynamics in toddlers remains limited, private speech is a visible marker of children beginning to guide their own attention and action with language—an everyday behavioral bridge toward the internally guided narrative/semantic integration emphasized in DMN accounts of older cognition (Smallwood et al., 2021; Menon, 2023).

##### 3.3.2.3 3–5 years: subsystem differentiation, narrative integration, and growing but effortful control

By the preschool years, DMN subsystems are more differentiated and more consistently coordinated with attention and control networks, although maturation remains ongoing (Xiao et al., 2016; Chen et al., 2021). This window is marked by expanding capacity for extended pretend-play scripts and multi-episode narratives, which place greater demands on maintaining and integrating information over longer temporal windows (Hasson et al., 2015; Lerner et al., 2011). Accordingly, engagement–integration rhythms can scale up: alongside brief restorative pauses, preschool contexts can support longer stretches of self-directed constructive play and project work, followed by narrative-rich reflection that encourages children to recount, re-sequence, and reinterpret experiences (e.g., story retell, collaborative planning-and-revision, emotion talk after challenging transitions). The key distinction from toddlerhood is not simply more “rest,” but richer integration demands—more complex, temporally extended content to integrate—and a greater role for child-generated narratives and self-explanation as tools for meaning-making. Accordingly, preschool routines should protect not only pauses after activity, but also opportunities for children to revisit, revise, and narratively reorganize extended experiences.

## 4 Discussion

This review supports a conservative thesis: DMN neuroscience is educationally relevant not because it prescribes narrow “brain-based” techniques, but because it clarifies constraints on how cognition alternates between external engagement and internal integration over time. Across the lifespan, DMN research converges on the idea that learning is not simply the accumulation of externally presented information, but also depends on internally oriented processes such as consolidation, integration with prior knowledge, narrative organization, and meaning construction (Menon, 2023; Smallwood et al., 2021). When interpreted cautiously, these constraints resonate with long-standing principles of early childhood practice—play-based learning, revisiting experiences, relational security, and time for reflection—while also helping explain why continuous, performance-driven task demands can be developmentally mismatched, particularly in the earliest years (Immordino-Yang, 2016).

The DMN should not be treated as a classroom target to be trained directly. Like other developing brain networks, it is embedded in a plastic developmental system whose connectivity and functional coordination are shaped over time by maturation, experience, sleep–wake state, stress physiology, social relationships, language, and opportunities for meaningful activity. As an illustrative application of the temporal-design argument, this conclusion is also relevant to contemporary early childhood contexts in which low-demand intervals and embodied play can be displaced by continuous digital stimulation; this point is treated here as contextual rather than DMN-specific, given evidence linking screen exposure to broader developmental outcomes and guidelines emphasizing physical activity, limited sedentary behavior, and adequate sleep in children under five (Hutton et al., 2020; Madigan et al., 2020; World Health Organization, 2019). Screen-based breaks may be enjoyable or useful in some contexts, but they should not be assumed to substitute for the low-demand conditions or vivid sensory experiences that allow children to turn inward, replay experience, construct narrative meaning, or engage in quiet imaginative thought.

From a conservative DMN-informed perspective, early childhood settings should protect not only sleep and physical rest, but also genuine wakeful rest and free play: screen-free or low-stimulation intervals, absorbed solitary, parallel, or collaborative play, quiet looking, drawing, reflective conversation, negotiations, and narrative revisiting. These recommendations do not depend on proving that such practices directly modify DMN connectivity; rather, they follow from converging evidence that learning and development require both externally guided participation and internally oriented integration over time.

Translation must remain deliberately modest. Educational neuroscience has been criticized for moving too quickly from neural observations to classroom prescriptions with many neuromyths being generated (Howard-Jones, 2014), and many DMN findings remain correlational and disproportionately adult-based. To reduce overreach, implications in this study were framed as (a) age-general design principles (rhythms of engagement and integration; narrative as infrastructure; emotionally secure contexts; constructive solitude within social ecologies) and (b) age-specific considerations that recognize shifting developmental constraints from infancy through preschool. This structure is intended to keep the level of inference appropriate: it does not claim that specific activities causally “build the DMN,” but rather that certain ecological conditions are coherent with how cognition toggles between externally and internally oriented modes, and with how network coordination is still stabilizing in early development.

## 5 Conclusion

The most defensible conclusion is pragmatic and protective. Early learning environments may benefit from safeguarding rhythms of external engagement and internal integration, maintaining abundant opportunities for narrative meaning-making, and preserving emotionally secure contexts in which children can revisit and interpret experience. In addition, classrooms can treat constructive solitude and self-directed activity as potentially developmentally meaningful rather than automatically problematic, while remaining attentive to forms of withdrawal associated with distress or exclusion (Coplan et al., 1994; Rubin et al., 2009). Because the DMN is embedded in a plastic developmental system, its connectivity and functional coordination should be understood as shaped over time by maturation, sleep–wake state, stress physiology, social relationships, language, and repeated meaningful activity. This plasticity does not justify activity-specific neural prescriptions, but it strengthens the rationale for protecting ecological conditions that allow young children to alternate between outward participation and inward integration.

## 6 Limitations and future research

Several limitations should be acknowledged. First, developmental neuroimaging imposes important state-related constraints on interpretation. Infant and toddler neuroimaging frequently relies on natural sleep acquisition, and sleep–wake state differences can alter functional connectivity estimates; developmental comparisons to primarily awake older-child or adult datasets therefore require caution (Mitra et al., 2017; Yates et al., 2023). In addition, internetwork measures—especially estimates of anticorrelation—can be sensitive to preprocessing and denoising choices (e.g., global signal regression) and to motion/physiological artifacts, which may vary systematically by age. Accordingly, claims about developmental changes in network “competition” should be interpreted as converging patterns rather than as single-pipeline certainties (Ciric et al., 2017; Murphy and Fox, 2017).

A further limitation concerns the uneven density of empirical evidence across the 0–6 window. While infancy and early toddlerhood are increasingly represented in resting-state datasets, the preschool period (approximately 3–5 years) remains comparatively under-studied in DMN-focused neuroimaging, partly due to practical and methodological constraints in acquiring high-quality data in awake young children (e.g., motion, state inference, age-appropriate preprocessing and parcellation). Accordingly, age-specific educational implications for the 3–5-year bracket should be interpreted as provisional and inference-based, relying on converging evidence from (a) adult/older-child DMN functional literature and (b) developmentally established behavioral findings, rather than direct preschool DMN intervention evidence. Future work should prioritize larger, awake preschool samples using child-friendly protocols (training/mock scanning, stringent motion handling), naturalistic paradigms, and complementary modalities (e.g., EEG/MEG/fNIRS) to better capture state transitions and ecologically valid learning contexts.

Third, much of the functional interpretation of the DMN is derived from adult studies, and adult-like spatial topography in young children does not entail adult-like function. Early childhood is a period of ongoing network reorganization and subsystem differentiation, so mapping neural patterns to educationally meaningful constructs must remain probabilistic and developmentally grounded rather than deterministic (Cao et al., 2017; Xiao et al., 2016).

Fourth, the interdisciplinary scope and aims of this work required conceptual synthesis rather than quantitative aggregation. As an integrative review, it is vulnerable to interpretive judgment, uneven evidence density across topics (e.g., stronger adult evidence than infant evidence for many DMN functions), and possible publication bias. In addition, screening and extraction were conducted by a single reviewer and the review protocol was not preregistered; therefore, selection and interpretive bias cannot be fully ruled out despite the use of predefined criteria, interval re-checking, and citation chaining.

Finally, translating neuroscience to education necessarily involves inference across levels of analysis. Causality cannot be assumed unless supported by intervention evidence, and pedagogical decisions must integrate ethical, cultural, and contextual considerations beyond neural plausibility. These limitations motivate the conservative translational stance adopted here: DMN science is used to clarify cognitive constraints and to interpret alignment with existing early childhood principles and practices, not to justify deterministic claims or activity-specific prescriptions.

Future research priorities include (a) naturalistic developmental neuroimaging that better captures ecologically valid learning states and mode switching, (b) longitudinal studies linking early network trajectories to later educationally relevant outcomes (with careful attention to sleep/wake state and motion confounds), and (c) intervention and design-based research testing whether protecting downtime, reflection, and narrative revisiting improves durable learning outcomes beyond immediate task performance. Progress will also require conceptual precision: differentiating beneficial solitude from distress-related withdrawal, and distinguishing rest, mind wandering, and reflective integration as potentially distinct internal states with different developmental functions.
